# Gender-Specific Disparities in Outcomes of Transcatheter Aortic Valve Implantation/Repair in Patients With Aortic Stenosis: A Systematic Review

**DOI:** 10.7759/cureus.111879

**Published:** 2026-07-01

**Authors:** Henry Cedeno, Ammara Akram, Anam Fatima Ansari, Belinda Eze, Mohammed Askari Meghji, Ayibongwe Sithole, Angelica Thomas, Siddharth Mitra, Nadeem Chauhan, Zainab Saleh, Tavsimran S Luthra, Sara Razi, Nipa Patel, Alina S Khan

**Affiliations:** 1 Medicine, Universidada de Guayaquil, Ecuador, ECU; 2 Medicine, Poonch Medical College, Rawalakot, PAK; 3 Medicine, Rama Medical College Hospital and Research Centre, Kanpur, IND; 4 Internal Medicine, St George’s University Hospital, London, GBR; 5 Medicine, University of Leicester, Leicester, GBR; 6 Medicine and Surgery, Princess Royal Hospital, London, GBR; 7 Medicine, Kingston Public Hospital, Jamaica, JAM; 8 Cardiology, Hinduhridayasamrat Balasaheb Thackeray Medical College, Mumbai, IND; 9 Medicine and Surgery, Oklahoma State University, Tulsa, USA; 10 Medicine, Hamad Medical Corporation, Doha, QAT; 11 Internal Medicine, Montefiore Medical Center, Wakefield Campus, Bronx, USA; 12 Medicine and Surgery, Islamic Azad University of Tehran, Tehran, IRN; 13 Medicine and Surgery, Om Sairam Cardiac Multispecialty Hospital, India, IND; 14 Medicine, Liaquat National Hospital and Medical College, Karachi, PAK

**Keywords:** aortic calcifications, aortic stenosis, cardiovascular adverse events, gender-specific disparities, tavr/tavi

## Abstract

Aortic stenosis caused by calcification and sclerosis of the left ventricular outflow tract is a life-threatening condition associated with a high risk of heart failure. Transcatheter aortic valve replacement (TAVR) is the definitive treatment for this condition. Gender-specific disparities influence post-procedural recovery, as women, despite generally having better long-term survival, have higher risks of short-term mortality, vascular complications, and major bleeding. Understanding these differences is essential for better post-operative care. We performed a comprehensive literature search of PubMed, Google Scholar, and the Cochrane Library using the keywords “Gender Disparities,” “TAVR,” “Aortic Stenosis,” and “TAVI.” Studies published between 2015 and 2025 were screened, yielding 25 eligible studies. Quality assessment was conducted using the Newcastle-Ottawa Scale for cohorts and the Cochrane risk-of-bias tool for randomized controlled trials. Overall, the included studies demonstrated moderate to high methodological quality, ensuring reliability of the findings. Female patients undergoing TAVR showed better long-term survival, with seven studies reporting higher all-cause mortality in females (p <0.05) and six reporting a significantly higher mortality in males (p<0.05). Stroke and MACE outcomes were mixed; some studies reported a higher incidence in females (p<0.05), whereas others found no significant difference (p > 0.05). Females had significantly higher rates of vascular complications, major or life-threatening bleeding (p<0.05), and a smaller aortic valve annulus. Secondary outcomes, including new pacemakers and non-home discharge, showed variable significance between genders. Women undergoing TAVR demonstrate better long-term survival despite higher rates of vascular complications and bleeding. Further research is needed to confirm these gender-related differences and guide post-procedural management.

## Introduction and background

Aortic valve stenosis (AVS) is the most prevalent valvular disease in the developed world, particularly among the elderly. AVS is caused primarily by progressive thickening and calcification, and less frequently by infectious scarring. Prognosis is poor at the onset of symptoms, with mortality exceeding 50% in untreated patients [1,2]. Procedures such as AVR (aortic valve replacement) and TAVR (transcatheter aortic valve replacement) have substantially increased survival [3]. Understanding sex-specific variations in clinical presentation and procedural outcomes is critical for optimizing patient selection and improving individualized treatment strategies in TAVR. Studies reveal that more than 40% of patients were never offered AVR during their lifetimes, reflecting persistent undertreatment [4,5]. Women exhibit a more gradual progression of AS, present at an older age, patients are more symptomatic and frail, they also tend to have higher morbidity and mortality, contributing to the rate of undertreatment [3,6]. Despite these established sex-related patterns, reported outcomes remain inconsistent across studies, likely due to evolving procedural techniques, valve generations, and patient selection criteria over time.

Several studies have suggested that female patients experience better clinical outcomes, with improved survival rates compared to men (76% vs. 65%, respectively) [7]. Female sex was independently associated with an increased risk of major vascular complications, but no differences for 30-day survival or stroke [8]. Other studies, at one-month follow-up, show that men exhibit a higher risk of post-TAVR stroke, vascular complications, and bleeding requiring transfusion [9]. In a meta-analysis conducted in 2013, which included 2706 men and 3178 women, mortality was higher in men compared to women (9.3% vs 6.9%), while the incidence of life-threatening bleeding higher in women (12.8% vs 15.3%), which was also applicable to major vascular complication (6.1% vs 11.3%), whereas stroke incidence was lower (2.2% vs 1.7%) [10]. In a separate study of 147,481 patients who underwent TAVR, one-month follow-up showed no sex differences in all-cause readmission or major adverse cardiovascular events (MACE) despite higher vascular complication rates in women (8% vs 4%) [8]. At six months, women had a higher risk of readmission for all causes, but a similar rate of hospital death [11]. Another study comprising 3013 men and 3649 women reported lower mortality in women compared to men (22.0% vs 16.9%). In general, women revealed more favorable short-, mid-, and long-term survival compared with men despite experiencing higher rates of major vascular complications, major bleeding events, and stroke [8,10].

Several studies have reported inconsistent results regarding sex disparities in outcomes following management with TAVR in patients with AS, including disparities in survival and mortality [12,13]. To address these inconsistencies, we systematically evaluated sex-specific outcomes following TAVR over the last decade using a comprehensive literature search across major medical databases. Two previous meta-analyses and systematic reviews were conducted in 2013 and 2023 [10,14]. Since then, several investigations have reported new outcomes, and trends have emerged. This systematic review integrates newly available data and recent trials to update and expand the existing literature, potentially redefining sex-based differences in TAVR outcomes. The results were synthesized to define homogeneous patterns of sex disparities and their clinical relevance for TARV outcomes [15].

## Review

Methods

This systematic review comprises 19 retrospective/prospective cohort studies and six randomized controlled/clinical trials [16-40]. The details are shown in Table 1. We conducted this research in accordance with the Preferred Reporting Items for Systematic Reviews and Meta-Analyses (PRISMA) guidelines and the *Cochrane Handbook for Systematic Reviews of Interventions* [41,42].

**Table 1:** Study Characteristics Study charcateritics of the randomized controlled trials and observational studies invcluded in this systematic reveiw. Abbreviations: MACE: Major adverse cardiac events, RCT: randomized controlled trials, TAVR: trans-catheter aortic valve repair, TAVI: trans-catheter aortic valve implant, CV: cardiovascular.

Study (Year)	Study Type	No. of patients	Intervention	Primary Outcomes	Secondary Outcomes	Follow-up Duration	Key findings
Pighi (2019)^16^	Prospective cohort	759	TAVR	12 months all-cause mortality	1 Month composite of mortality or major morbidity, discharge to a non-home setting, or rehabilitation facility.	12 months	Women were more likely to require discharge to a rehabilitation facility. The risk of 1 - month mortality or major morbidity was greater in women particular those treated with large prostheses
Kumar et al. (2025)^17^	Retrospective cohort	320,324	TAVR	In-hospital mortality, stroke, vascular complications, readmission rates at 30 days and 90 days.	Post-procedural bleeding, pericardial complications, acute respiratory failure (ARF), need for transfusion, need for vasopressors, and major adverse cardiac and cerebrovascular events (MACCE), acute kidney injury (AKI), sudden cardiac arrest (SCA), cardiogenic shock, and mechanical circulatory support.	Up to 180 days	Women had higher mortality and post-procedural complication rates. Men had higher readmission rates, cardiogenic shock, AKI, and need for mechanical circulatory support
See et al. (2024)^18^	Retrospective cohort	927	TAVI	Mortality scores 1-year.	Cardiovascular comorbidities include coronary artery disease, peripheral artery disease, and smoking. Size of valves	1 year	No significant difference in 1 year survival between sexes using the primary new generation valve
Nakase et al. (2024)^19^	Prospective cohort	2,026	TAVR	All-cause mortality	Mortality by stages of cardiac damage	5 years	In the early stages of Cardiac damage Women had a lower 5-year mortality than men. In more advanced stages, mortality was comparable between sexes
Chaker et al. (2017)^20^	Retrospective cohort	166,809	TAVR	In‐hospital mortality, rates of stroke, length of hospital stay, and rates of non-home discharge	Vascular complications, blood transfusion, permanent pacemaker implantation, and acute kidney injury requiring dialysis.	30 days	Woman higher in-hospital mortality following AVR, and more instances of vascular complications and blood transfusion. Rates of stroke, permanent pacemaker implantation, and acute kidney injury were the same in both groups. Length of stay was the same in both groups. Rates of non-home discharge were higher among women
Wang et al. (2019)^21^	Prospective cohort	298	TAVI with THV 's	Hospital length of stay and in-hospital rates of mortality	Aortic annular area, disabling stroke, and pacemaker.	30 days	No significant difference in clinical outcomes in men and women
Kim et al. (2024)^22^	Prospective cohort	1,412	TAVR	All-cause mortality, stroke, and size of the aortic valve	Length of hospital stay	1 Year	No sex specific differences were seen between the two groups, and all-cause mortality, but a higher risk of stroke was seen more in males
Thevathasan et al. (2025)^23^	RCT	299	TAVI	All-cause mortality, discharge to a non-home setting, and the size of the aortic valve	New pacemaker, acute kidney injury, MACE, pericardial complications, and bleeding	90 Days	Women were more likely to be discharged to a non-home facility, and female patients had more post-procedural complications
He et al. (2022)^24^	Retrospective cohort	510	TAVR	Vascular complications, bleeding, and all-cause mortality	Pericardial complications, stroke, and MACE	1 year	No difference in 1 year mortality between the two groups, but male patients had fewer in-hospital complications
Jhonston et al. (2024)^25^	Prospective cohort	14,123	AVR	All-cause mortality, stroke, and bleeding	N/A	8 years	No significant long-term difference in CV events between men and women
Bière et al. (2015)^26^	prospective cohort	3,972	TAVR	All-cause mortality, size of aortic valve annulus, and major bleeding	New pacemaker and acute kidney injury	1year	Women exhibited higher post-procedural adverse events
Chandrasekhar et al. (2016)^27^	prospective cohort	23,652	TAVT	All-cause mortality and MACE	Stroke and bleeding	1 year	Women had higher risk scores for the primary outcomes
Chang et al. (2020)^28^	prospective cohort	221	TAVI	All-cause mortality and the aortic valve annulus	MACE, new pacemaker, acute renal failure, and stroke	1 year	A smaller aortic valve is used in females, and higher all-cause mortality in females
Du et al. (2018)^29^	Retrospective cohort study	73	TAVR	All-cause mortality, stroke, and size of the aortic valve	New pacemaker	30 days and 1 year	Women had a significantly smaller aortic valve annulus, no difference between women and men in all-cause mortality, and LVEF at discharge was higher in women
Singh (2019)^30^	Prospective cohort study	674	Ventricular remodeling and TAVI	All-cause mortality and size of the aortic annulus	Peri-procedural complications	6 years	No significant difference observed
Van Meigham (2020)^31^	RCT	1,660	TAVI	All-cause mortality, stroke at two years, and major bleeding	New pacemaker and pericardial complications	2 years	Female patients showed higher mortality and major bleeding complications compared to men
Vlastra (2019)^32^	RCT	12,381	TAVR	All-cause mortality	Post-procedural bleeding, readmissions, new pacemaker, stroke, and vascular complications	30 days and 11 years	No specific difference noted in post-procedural outcomes
Czarnecki (2017)^33^	prospective cohort	999	TAVR	All-cause mortality	All cause readmission	3.5 years	No significant difference was noted between the two groups
Forrest (2016)^34^	prospective cohort	3687	TAVR	All-cause mortality at 30 days and at 1 year, size of the aortic valve	Incidence of stroke at 30 days and 1-year, major vascular complications, MACE, and acute kidney injury	1 year	Women experienced more all-cause mortality, stroke, and bleeding post-procedure
Kaier (2018)^35^	prospective cohort	9,474	TAVR	All-cause mortality, stroke, and vascular complications	Post-procedural bleeding	1-2 years	Female patients had a lower risk of stroke and all-cause mortality, but more bleeding
Katz (2017)^36^	prospective cohort	819	TAVI	All-cause mortality, vascular complications, and major bleeding	Periprocedural complications	30 days	Female patients exhibited higher periprocedural complications
Kodali (2016)^37^	RCT	2559	TAVR	All-cause mortality, major vascular complications, stroke	Paravalvular leak, new pacemaker, acute renal failure	30 days and 1 year	Women had a higher all-cause mortality and vascular complications
Greener (2018)^38^	prospective cohort	150,647	TAVR	All-cause mortality, major life-threatening bleeding	Vascular complication. Paravalvular leak, new pacemaker implantation, and acute renal failure	6 years	No difference noted in most outcomes. Female patients had higher in-hospital mortality compared to male patients
Szerlip (2016)^39^	RCT	1661	TAVR	Aortic valve size, all-cause mortality	Major vascular complications and bleeding	30 days/ 1 year	No sex-specific differences in survival or stroke following TAVR. Female patients had a higher incidence of vascular complications
Worhle (2022)^40^	RCT	1644	TAVI	All-cause mortality, MACE	Non-cardiovascular mortality	17 months	Female patients had a lower risk of MACCE and all-cause mortality, but no difference in the risk of bleeding events after TAVI

Study Registration

The aim of this systematic review was to assess the differences in outcomes between men and women after TAVR for severe aortic stenosis, regardless of etiology. The protocol was formally registered in the PROSPERO database (Registration ID: CRD420241147228) prior to study initiation. All methodological components, including the search strategy, eligibility criteria, study selection process, and data extraction methods, were predefined to ensure transparency, reproducibility, and minimization of bias throughout the review process.

*Search Strategy and Study Selection* 

To provide a comprehensive and contemporary assessment, we systematically evaluated sex-specific outcomes in patients with aortic stenosis undergoing transcatheter aortic valve replacement (TAVR) over the past decade and updated the existing literature by incorporating six newly published studies. A structured search was conducted across five major databases: PubMed, Google Scholar, Embase, Scopus, and the Cochrane Library, selected for their broad coverage of randomized controlled trials, observational studies, and large registry-based analyses.

The search spanned January 2015 to October 2025 and was performed using a combination of Medical Subject Headings (MeSH) and free-text keywords. Search terms related to the study population, intervention, and outcomes of interest included “aortic stenosis,” “severe aortic stenosis,” “transcatheter aortic valve implantation,” “transcatheter aortic valve replacement,” “TAVI,” “TAVR,” “sex differences,” “gender disparities,” “sex-specific outcomes,” “mortality,” “stroke,” “bleeding,” “vascular complications,” and “postoperative outcomes.” These terms were combined using Boolean operators (“AND” and “OR”) and adapted according to the indexing requirements of each database. In addition, the reference lists of included studies and relevant review articles were manually screened to identify any potentially eligible studies not captured through the electronic database search. The complete search strategy is illustrated in Supplementary Table S1.

Data Extraction Process

Study screening was conducted independently by two reviewers using Rayyan (Rayyan Systems Inc., Cambridge, MA, USA), an AI-assisted systematic review tool that enables blinded screening, automatic deduplication, and qualitative study selection to ensure systematic screening and minimize selection bias. After carefully examining the inclusion criteria, all relevant data were organized onto a standardized Microsoft Excel (Microsoft, Redmond, WA) extraction sheet. Extracted variables included author name, publication year, study design, total number of patients, baseline patient characteristics, primary and secondary outcomes, follow-up duration, and key findings for each study.

Following data extraction, study characteristics and outcomes were synthesized descriptively to compare clinical outcomes between female and male patients undergoing TAVR. Any discrepancies between reviewers were resolved through discussion or, if consensus could not be reached, by a third reviewer to ensure accuracy and consistency. Only peer-reviewed, full-text articles were included in the final analysis to ensure methodological rigor.

Eligibility Criteria

Inclusion Criteria

This systematic review employed a rigorous methodology focused on selecting high-quality and clinically relevant studies. Potentially eligible articles underwent full-text review and were assessed against predefined inclusion criteria. Studies were included if they: (1) enrolled adult patients with severe aortic stenosis, irrespective of etiology, undergoing TAVR; (2) reported outcomes for both women and men or provided sufficient data for sex-based comparisons; and (3) evaluated at least one clinically relevant outcome, including all-cause mortality, cardiovascular mortality, stroke, bleeding events, vascular complications, rehospitalization, functional status, quality of life, or other major adverse cardiovascular outcomes. Randomized controlled trials, prospective cohort studies, retrospective cohort studies, and large registry analyses were considered eligible. Only peer-reviewed full-text studies published within the predefined search period were included.

Exclusion Criteria

Studies that did not meet the inclusion criteria were excluded from this review. Case reports, case series, review articles, systematic reviews, meta-analyses, conference abstracts, editorials, letters to the editor, and website articles without available full manuscripts were not included. Studies that did not report sex-specific outcomes or did not provide sufficient data to allow a comparison between women and men were excluded. Publications evaluating surgical aortic valve replacement (SAVR) alone, studies involving animal models or in vitro data, and studies involving mixed valvular populations in which outcomes for isolated aortic stenosis or TAVR could not be separated were also excluded. Non-English language studies without an available English translation and duplicate publications from the same study population were excluded from the final analysis.

Risk of Bias and Quality Assessment

Two widely recognized tools were used to assess the quality of the studies incorporated in this review. The Newcastle-Ottawa scale (NOS) was employed for observational studies [43]. This tool assesses quality across three domains: (i) selection of study participants, (ii) comparability of study groups, and (iii) ascertainment of exposure or outcomes. Each aspect is scored with stars, with a maximum of nine. A higher score indicates less potential for bias, while a lower score suggests possible weaknesses. Studies scoring 7-9 were considered high quality, 4-6 were moderate, and 0-3 were low [44]. The quality assessment of the included cohort studies in this paper shows that most studies scored between 6 and 9, suggesting moderate to high methodological quality. Key limitations included comparability and follow-up, with some studies lacking sufficient baseline outcome evaluation. The majority of studies met adequate selection criteria and outcome evaluation, confirming the quality of their results as seen in Table 2.

**Table 2:** New Castle Ottawa (NOS) Scale, Quality Assessment Quality evaluation of observational studies via New Castle Ottawa (NOS) Scale for cohort studies

Study	Selection (score）				Comparability (score)		Outcome(score)			Total Score	
												representativeness of the exposed cohort	Selection of the non-exposed cohort	Ascertainment of exposure	Demonstration that outcome of interest was not present at start of study	select the most important factor	Any additional factor	Assessment of outcome	Follow-up period	Adequacy of follow up of cohorts	
											Pighi (2019) ^16^	1	1	1	1	1	1	1	1	1	9	
Kumar (2025)^17^	1	1	1	1	1	1	0	1	1		8	
See (2024)^18^	1	1	1	1	1	0	0	1	1	7	
Nakase (2024)^19^	1	1	0	1	1	0	1	1	0	6	
Chaker (2017)^20^	1	1	1	1	1	1	1	1	0	8	
Wang (2019)^21^	1	1	1	1	1	0	1	1	0	7	
Kim (2024)^22^	1	1	1	0	1	1	1	1	1	8	
He (2022)^24^	1	1	0	1	0	1	1	1	0	6	
Jhonston (2024)^25^	1	1	1	1	1	1	1	1	1	9	
Biere (2015)^26^	1	1	0	1	1	1	1	1	0	7	
Chandrasekhar (2016)^27^	1	1	1	1	1	1	0	1	1	8	
Chang (2020)^28^	1	1	1	0	0	1	0	1	1	6	
F.Du (2018)^29^	1	1	1	1	1	1	1	1	1	9	
Singh (2019)^30^	1	1	1	0	1	1	1	1	1	8	
Czarnecki (2017)^33^	0	1	1	1	1	1	0	1	0	6	
Forrest (2016)^34^	1	1	0	1	1	1	1	0	1	7	
Kaier (2018)^35^	1	0	1	1	1	1	1	1	1	8	
Katz (2017)^36^	0	1	1	0	1	1	1	1	1	7	
Greener (2018)^38^	1	1	1	1	1	1	1	1	1	9	

**Figure 1:**
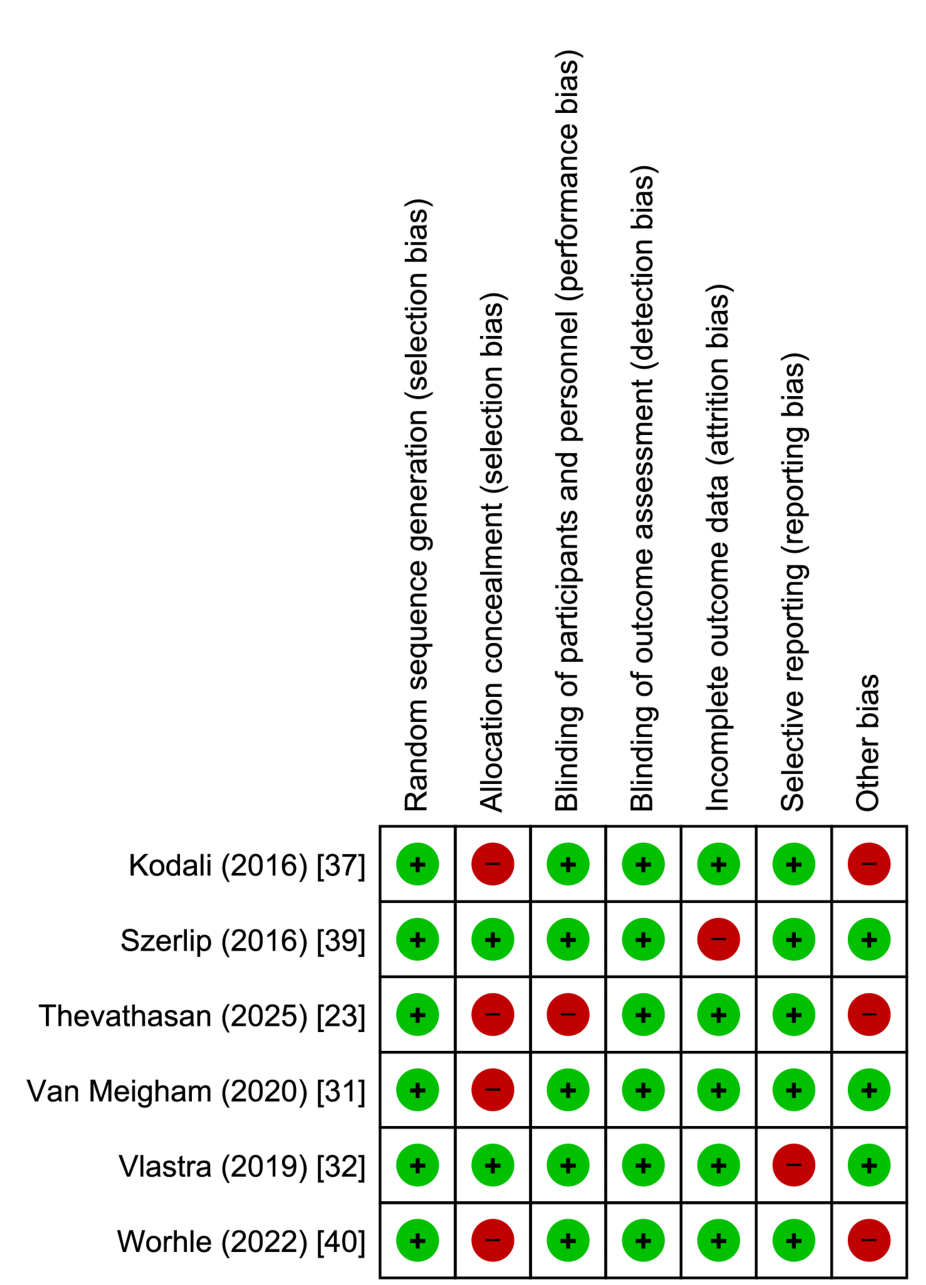
Risk of Bias Summary Risk of bias summary as part of the quality assessment of randomized controlled trials.

**Figure 2:**
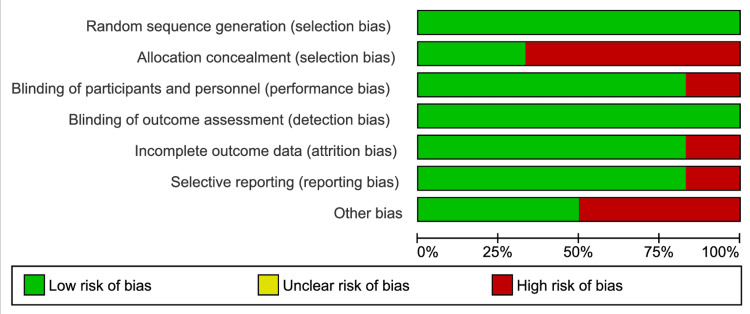
Risk of Bias Graph Risk of bias graph depicting the quality assessment of randomized controlled trials.

For randomized controlled clinical trials (RCTs), the Cochrane Risk of Bias Tool was used. This instrument evaluates several domains, including random sequence generation, allocation of concealment, blinding of participants and investigators, blinding of outcome assessment, incomplete outcome data, and selective reporting. Each domain is judged to have low, high, or unclear risk of bias [45]. The majority of randomized trials in this systematic review demonstrated a low risk of bias, as assessed across the following parameters: random sequence generation, blinding of outcome assessment, and handling of attrition. Due to difficulties in blinding participants and staff, allocation concealment, selective reporting, and performance bias showed high risk. The methodological quality of the included trials was generally good, with blinding and reporting domains being the primary areas of constraint. This is illustrated in Figure 1 (risk of bias summary) and Figure 2 (risk of bias graph). By combining these approaches, we ensured that both observational and randomized evidence were systematically evaluated. This allowed us to assess the data quantitatively and qualitatively, based on the strength and reliability of the findings in our review.

Results

Study Characteristics

**Figure 3:**
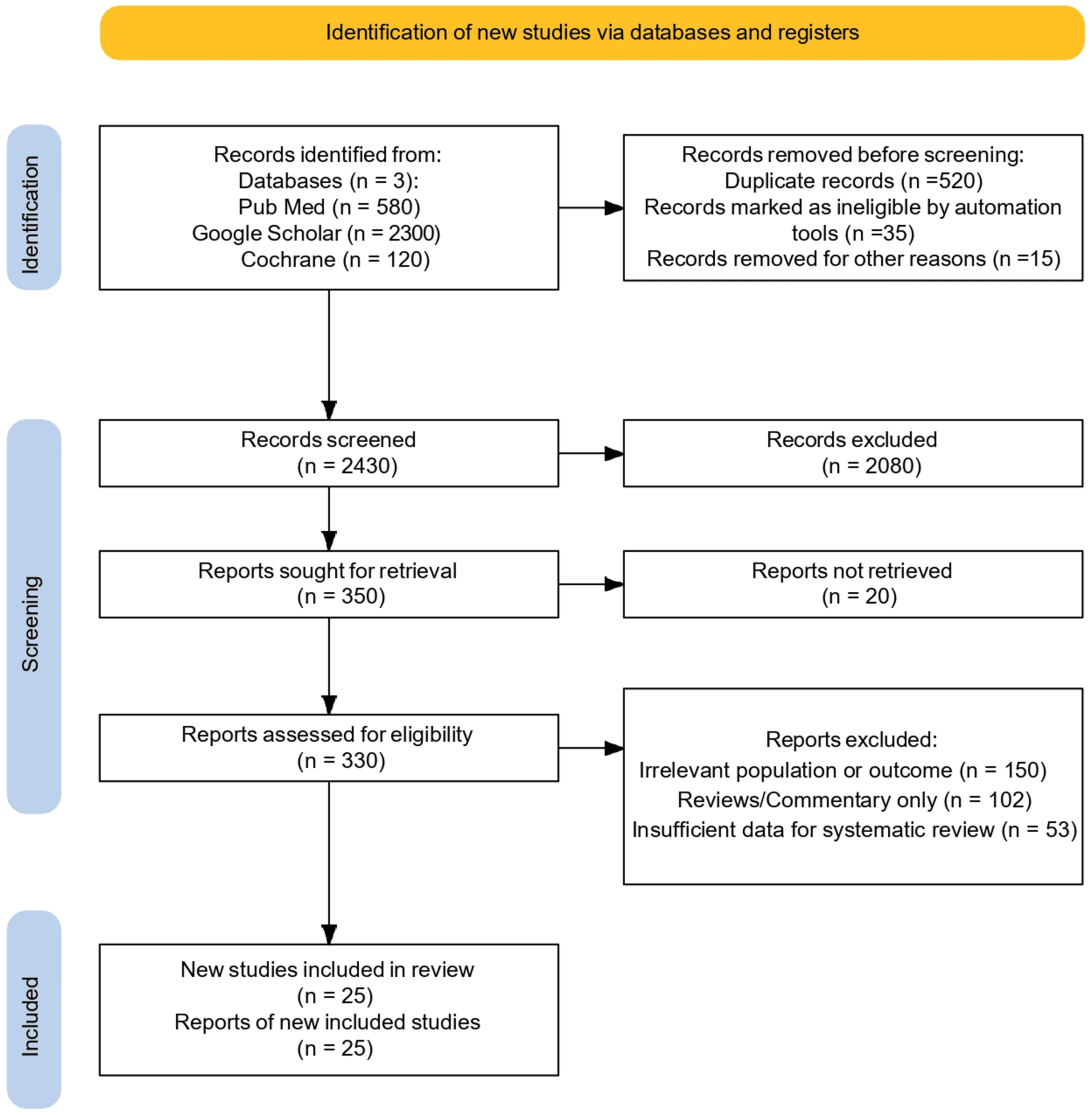
PRISMA Flow Chart of Selected Studies The flow diagram shows the number of records identified, screened, excluded, and included in the systematic review. PRISMA: Preferred Reporting Items for Systematic Reviews and Meta-Analyses

Initially, our search yielded 3000 studies: 580 articles were retrieved from PubMed, 2300 from Google Scholar, and 120 from the Cochrane Library. After deduplication and exclusion of articles that did not meet our inclusion criteria, 2430 records were screened for titles and abstracts. After a comprehensive review of the records, 330 studies were considered for full-text assessment. A further 305 studies were excluded, leaving 25 included in this systematic review. The data is displayed in Figure 3 (Preferred Reporting Items for Systematic Reviews and Meta-Analyses (PRISMA) flow chart).

Synthesis of Results

**Figure 4:**
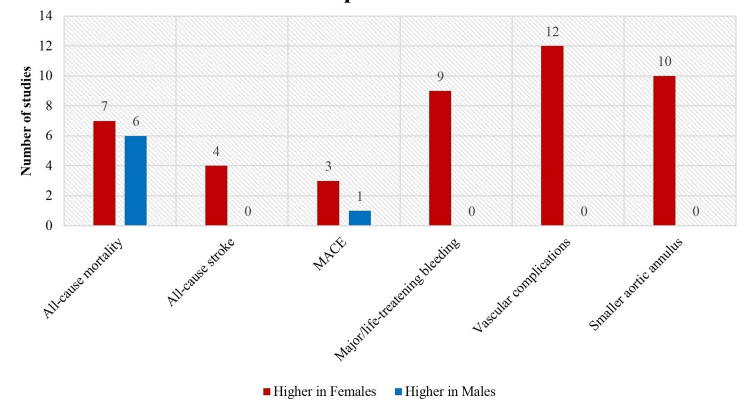
Distribution of Studies Reporting Significant Primary Outcomes. This bar chart illustrates the distribution of studies that only reported statistically significant (p < 0.05) gender-based differences in primary outcomes following TAVR/TAVI. MACE: Major Adverse Cardiovascular Events

The primary outcomes of interest analyzed in our review were: all-cause mortality, all-cause stroke, MACE, major/life-threatening bleeding, vascular complications, and the size of the aortic valve annulus. The studies demonstrating a significant association between gender and post-procedure complications are illustrated in a bar graph in Figure 4, and the summary of all primary outcomes reported by each study is shown in Table 3.

**Table 3:** Summary of Primary Outcomes *Significant outcomes with a reporting p-value<0.05.

Outcome	Studies Reporting Outcome (n)	Higher in Females	Higher in Males	No Significant Difference
All-cause mortality	25	7 studies (7 significant*)	7 studies (6 significant; 1 non-significant)	11 studies
All-cause stroke	22	6 studies (4 significant*)	2 studies (not significant)	14 studies
Major adverse cardiovascular events (MACE)	11	3 studies (3 significant)	1 study (significant*)	7 studies
Major / life-threatening bleeding	17	11 studies (9 significant*)	0	6 studies
Vascular complications	15	12 studies (12 significant*)	0	3 studies
Aortic valve annulus size	11	10 studies (all significant*; smaller in females)	0	1 study

All-Cause Mortality

All 25 recruited studies reported the outcome, all-cause mortality in patients with aortic stenosis undergoing TAVR. Seven studies found a significantly (p<0.05) higher all-cause mortality in the female group as compared to the male group. Another seven studies reported higher all-cause mortality in male patients; six were statistically significant, while one was non-significant (p>0.05). The remaining 11 studies did not identify a statistically significant difference between the two genders.

All-Cause Stroke

All-cause stroke was reported as a primary outcome by 22 out of the 25 recruited studies. Six studies demonstrated a higher incidence of stroke in female patients compared with male patients; four of these studies reported statistically significant findings (p<0.05). Two studies showed a higher incidence of all-cause stroke in the male demographic compared to the female, but the data were statistically insignificant (p>0.05). The remaining 14 studies did not report a significant difference between the two groups.

*Major Adverse Cardiovascular Events* (*MACE*)

Major adverse cardiovascular events following TAVR, including myocardial infarction (MI), heart failure, arrhythmias, or sudden cardiac death, were evaluated as an outcome of interest in 11 studies. Three studies reported a higher incidence of MACE among female patients, all of which were statistically significant (p<0.05). One study observed a higher incidence in men, which was also statistically significant. The remaining studies reported no significant difference between male and female patients.

Major/Life-Threatening Bleeding

This outcome entails any incidence of major or life-threatening bleeding episodes that occurred after the TAVR procedure in patients with aortic stenosis. Seventeen studies reported this adverse event; 11 demonstrated a higher incidence of post-procedure bleeding in the female population compared to the male population, of which nine reported statistically significant results (p<0.05). The remaining six studies found no significant gender differences between the two groups.

Vascular Complications

Vascular complications after TAVR were reported in 15 studies. Twelve studies revealed that females had a significantly (p<0.05) higher rate of vascular complications compared with males. The remaining three reported no significant gender disparity in this outcome.

Size of the Annulus of the Aortic Valve

The size of the aortic valve annulus used during the procedure was analyzed by 11 studies. Ten studies demonstrated a significantly (p<0.05) smaller aortic valve annulus in female patients compared to male patients. One study reported a statistically insignificant (p>0.05) difference. The use of a smaller valve increases the chances of vascular complications, which proves that female patients were at a significantly higher risk of post-procedure adverse events compared to the male patients.

Secondary Outcomes

Several secondary outcomes were considered for this systematic review. Thirteen studies examined the need for a new pacemaker following TAVR. Five of these reported statistically significant findings, with four demonstrating a higher incidence in male patients. Ten studies evaluated pericardial complications; three exhibited a higher incidence in female patients, while one reported a higher incidence in male patients. Five studies reported hospital readmissions; two observed significantly higher rates in male patients than in female subjects. The length of hospital stay after a TAVR procedure was analyzed in six studies; only three reported a significantly longer stay in female patients and a shorter stay in male patients. Ten papers evaluated the incidence of acute kidney injury post-operatively, but only three reported a statistically significant correlation in the male group. Discharge to a non-home facility after the procedure was reported in only three studies, and female patients were more likely than the male group to be sent there, a finding that was statistically significant. Five studies observed a need for transfusion and vasopressors post-procedure; two of which demonstrated statistically significant requirements in the male group, and only one paper reported a greater need for transfusions and vasopressors in the female group. The remaining studies did not report a statistically significant difference in any secondary outcome.

Discussion

Aortic stenosis (AS) is a narrowing of the aortic valve caused by calcific degeneration in most cases in developed nations [46]. The prevalence of AS increases with age, rising steadily from about age 65 and with a slight predominance in men [47,48]. Guidelines exist for the management of valvular diseases [49]. However, in general, aortic valve replacement is considered in severe symptomatic AS, typically SAVR [50].

Transcatheter aortic valve replacement (TAVR) is a minimally invasive procedure that is used to treat severe aortic stenosis as an alternative to SAVR in patients considered high risk [51]. In TAVR, a new prosthetic valve is delivered to the aortic root via a catheter, most commonly through the femoral artery or via alternative routes such as transapical or trans-aortic [51]. The new prosthetic valve is then expanded within the annulus of the native valve, which is believed to fragment the calcific deposits, thereby improving flow through the aortic valve [52].

There has been a considerable amount of literature published investigating the gender disparities in patients undergoing TAVR with a wide range of outcomes. A study conducted by D’Ascenzo et al. found that while overall mortality remained similar, major vascular complications and overall bleeding remained significantly higher in women [53]. In another study, while comparing the baseline data, women were found to be older and have increased frailty, but they presented with lower co-morbidities, higher left ventricular systolic function, and less coronary artery disease. The study concluded the same, with women having a higher incidence of major vascular complications and even stroke [34]. In another study conducted across tertiary hospitals in Canada, it was demonstrated that despite women having worse vascular outcomes, female sex is associated with better short-term (30 days) survival after TAVR [54]. In a long-term study, Klodas et al. found that the 10-year survival rate was lower in women than in men. In multivariate regression, female sex was an independent predictor of worse late survival [55]. However, some studies found that without propensity-matching for baseline characteristics and perioperative variables, overall comorbidity was higher in women than in men; after adjustment, there was no statistically significant difference [56]. Our review of eligible studies offers a broader, more comprehensive overview of this association by incorporating recent research conducted from October 2023 to September 2025.

The target population for this research was patients with aortic stenosis undergoing TAVR; female patients comprised the intervention group, and male patients comprised the comparator group. This systematic review indicates that gender disparity is an independent factor that influences the trajectory toward successful TAVR. These findings could be because of various reasons. Women often present with more frequent co-existent moderate/severe mitral valve disease, New York Heart Association (NYHA) functional class III/IV symptoms. Studies have found women to be older and have more comorbidities, which negatively impact outcomes [57]. Men, on the other hand, exhibited an increased incidence of preexisting comorbidities compared to women, which included diabetes mellitus, previous myocardial infarction, and previous percutaneous coronary intervention [57]. Despite these other factors, females differ anatomically from males, which affects the trajectory of post-op recovery. They have a narrower annulus, which requires a smaller prosthesis and increases the likelihood of prosthesis-patient mismatch and vascular injury or accidents during the procedure [58].

Our study consisted of a systematic review of 25 articles published in PubMed, Google Scholar, and the Cochrane Library, selected after a comprehensive screening to meet our inclusion criteria. In this research, we focused on the following outcomes: all-cause mortality, all-cause stroke, vascular complications, MACE, major/life-threatening bleeding, and the size of the aortic valve annulus.

This systematic review evaluated the differences in gender-based outcomes after TAVR. The main findings of our study highlighted that although women undergoing TAVR often present older, with higher procedural risk and smaller annular dimensions, they tend to have comparable all-cause mortality, stroke, and MACE compared with men. Previous studies have shown favorable long-term mortality outcomes in women compared to men [14]. All 25 recruited studies reported all-cause mortality as a primary outcome; seven of these demonstrated a statistically significant (p-value<0.05) risk of post-procedure mortality at 30 days and at the 10-year mark in the female group compared to the male group. Another seven studies reported higher all-cause mortality in the male group, and the remaining 11 studies did not show a significant difference (p>0.05). Similarly, for all-cause stroke, most studies did not report significant gender disparities despite some trends (four out of 22 studies) towards higher incidences in female patients. A 2025 sub-analysis of an RCT showed comparable ischemic and bleeding outcomes 30 days after TAVR in patients on oral anticoagulation [59]. A similar finding was noted in another cohort study evaluating 511 patients with 30 days post-TAVR, major or life threatening bleeding was reported by 17 studies and 11 of these exhibited a statistically significant incidence of post-procedure bleeding in the female demographic compared to the male population; moreover, the data collected from each study displayed a p value less than 0.05 instantly making this event an outcome of interest [60].

Numerous studies highlighted that the implementation of ultrasound-guided access, pre-procedural CT-based vascular mapping, and lower-profile sheaths could significantly mitigate this disparity, an essential clinical consideration that should influence peri-procedural planning [61]. Twelve of the 15 studies that reported major vascular complications showed p-values less than 0.05, indicating that the outcome had a statistically significant impact on the overall trajectory of the research. Most of the findings are consistent with the previous meta-analysis conducted in 2023, but the new studies from 2023 to 2025 added newer data on the number of patients reporting on the outcomes [14]. The higher incidence of vascular complications in the female gender could be attributable to smaller ilio-femoral vessel diameters, which increase the sheath-to-artery ratio during transfemoral access, leading to more complex device manipulation and vascular injury [24].

For MACE, our findings were mixed. Only four out of 11 studies showed significant (p<0.05) gender-related disparities (three in female patients and one in male patients). This review evaluated MACE as any clinical episodes of MI (myocardial infarction), heart failure, arrhythmia, and sudden cardiac death, all of which are influenced by multiple factors such as baseline comorbidities and procedural techniques such as anticoagulation. The lack of consistency across studies also suggests that other factors unrelated to gender are at play, as van Bergeijk suggests [58]. Another important outcome was the size of the aortic valve annulus used for the TAVR procedure. The differences in valve anatomy between the two groups play a major role in post-procedure recovery and rehabilitation [12]. This systematic review considered the size of the aortic valve annulus as the primary outcome. Eleven of the recruited research papers reported that women required a narrower prosthesis than men. Given the possible complications associated with a smaller annulus, female patients showed a worse post-procedure response than male patients, and the p-values reported in these 10 studies were less than 0.05, indicating this outcome was statistically significant.

This research paper also demonstrated secondary outcomes, which showed some gender related patterns, such as pericardial complications, primarily, pericardial effusion/cardiac tamponade, are an uncommon complication following TAVR. We found increased findings in women, consistent with a cohort study of 1247 patients and a post hoc analysis of an RCT that also reported similar findings [13,31]. These findings could be attributed to aortic annulus rupture, especially with small annuli, balloon-expandable valves, and stiff guide wires used for valve delivery, resulting in left ventricular rupture. Primary pacemaker implantation (PPI) is a recognized complication following TAVR. This occurs due to disruption of the cardiac electrical pathway, resulting in heart block and necessitating a PPI. Overall, men had higher PPI rates than women. Female patients were more likely to have a longer hospital stay than male patients. Some studies also demonstrated that women were more likely to be discharged to non-home facilities such as skilled nursing homes, due to the unstable nature of their post-operative condition. This study shows that during TAVR, awareness of the higher risk of vascular complications in women must be taken into account. Additionally, female patients also displayed a higher rate of readmission to the hospital, increased incidence of acute kidney injury, and increased use of vasopressors or transfusion after the TAVR procedure.

Recent research indicates that device selection, especially the use of contemporary low-profile transcatheter heart valve (THV) systems, may affect outcomes for transcatheter aortic valve replacement (TAVR) patients by gender. The Evolut FX and Sapien 3 Ultra Resilia are two recent devices that may help gradually reduce the gap in TAVR outcomes between men and women by minimizing complications [62].

Strengths and limitations

Our study used a large, aggregated sample of over a thousand patients, pooling data to improve our statistical understanding of sex-related disparities in TAVR outcomes. Our study synthesized findings from diverse sources, including single-center and registry studies, as well as specific randomized controlled trials (RCTs) and retrospective/ prospective cohort studies. The study also assessed multiple clinically relevant outcomes, providing a more balanced perspective on efficacy and outcomes. We also used the Newcastle-Ottawa Scale to assess the risk of bias in observational studies and the Cochrane Risk of Bias tool 2.0 for RCTs, further reducing bias and making our study more relevant. This review compiled data from 2015 to 2025, providing a larger cohort of patients and strengthening the overall results of each study. Data collection was conducted in accordance with the 2020 PRISMA criteria, ensuring a comprehensive and credible literature search. This study is also registered with PROSPERO.

However, our study also had some limitations: (i) Heterogeneity across studies due to differences in device type, baseline patient risk profile, and comorbidity burden; it often reduces confidence in the effect size. (ii) Potential publication bias where smaller studies showing no differences may be under-represented. (iii) Short follow-up in some studies (30-day or one-year outcomes) makes it difficult to obtain a collective understanding of long-term outcomes (beyond five years). (iv) Limited understanding of socioeconomic and referral disparity, which includes access to care, timing of referral, and symptom recognition. Further research is needed to properly understand why women face higher early complications, to improve patient care, and prevent the occurrence of post-procedure adverse events.

From a clinical perspective, our findings support sex-specific considerations in TAVR planning, particularly regarding access route selection, device choice, vascular imaging, and post-procedural monitoring.

## Conclusions

The systematic review demonstrated that women undergoing TAVR had significantly higher rates of bleeding and vascular complications, along with increased overall mortality, compared to men. These findings suggest that female patients with aortic stenosis undergoing TAVR are at a greater risk of adverse procedural and post-procedural outcomes. Potential contributors include smaller vascular anatomy, differences in comorbidity profiles, and sex-specific physiological factors. Early recognition of these risks is essential to inform pre-procedural planning, optimize intraoperative strategies, and enhance post-procedural monitoring, ultimately improving outcomes and minimizing complications in both sexes.

## Appendices

**Table 4:** Supplementary table S1 Search Strategy

Database	Search Strategy	Number of articles found
PubMed	("male"[MeSH Terms] OR "male"[All Fields] OR ("femal"[All Fields] OR "female"[MeSH Terms] OR "female"[All Fields] OR "females"[All Fields] OR "female s"[All Fields] OR "femals"[All Fields])) AND ("outcome"[All Fields] OR "outcomes"[All Fields]) AND (("patient s"[All Fields] OR "patients"[MeSH Terms] OR "patients"[All Fields] OR "patient"[All Fields] OR "patients s"[All Fields]) AND ("aortic valve stenosis"[MeSH Terms] OR ("aortic"[All Fields] AND "valve"[All Fields] AND "stenosis"[All Fields]) OR "aortic valve stenosis"[All Fields] OR ("aortic"[All Fields] AND "stenosis"[All Fields]) OR "aortic stenosis"[All Fields])) AND (("aortic valve"[MeSH Terms] OR ("aortic"[All Fields] AND "valve"[All Fields]) OR "aortic valve"[All Fields]) AND ("dysfunctional"[All Fields] OR "dysfunctional"[All Fields] OR "dysfunctioning"[All Fields] OR "dysfunctions"[All Fields] OR "physiopathology"[MeSH Subheading] OR "physiopathology"[All Fields] OR "dysfunction"[All Fields])) AND ("trans"[All Fields] AND ("catheter s"[All Fields] OR "catheters"[MeSH Terms] OR "catheters"[All Fields] OR "catheter"[All Fields]) AND ("aortic valve"[MeSH Terms] OR ("aortic"[All Fields] AND "valve"[All Fields]) OR "aortic valve"[All Fields]) AND ("replace"[All Fields] OR "replaceable"[All Fields] OR "replaced"[All Fields] OR "replaces"[All Fields] OR "replacing"[All Fields] OR "replacment"[All Fields] OR "replantation"[MeSH Terms] OR "replantation"[All Fields] OR "replacement"[All Fields] OR "replacements"[All Fields])) AND ("trans"[All Fields] AND ("catheter s"[All Fields] OR "catheters"[MeSH Terms] OR "catheters"[All Fields] OR "catheter"[All Fields]) AND ("aortic valve"[MeSH Terms] OR ("aortic"[All Fields] AND "valve"[All Fields]) OR "aortic valve"[All Fields]) AND ("repairability"[All Fields] OR "repairable"[All Fields] OR "repaire"[All Fields] OR "repaired"[All Fields] OR "repairment"[All Fields] OR "wound healing"[MeSH Terms] OR ("wound"[All Fields] AND "healing"[All Fields]) OR "wound healing"[All Fields] OR "repair"[All Fields] OR "repairing"[All Fields] OR "repairs"[All Fields]))	580
Google Scholar	(''Trans Catheter aortic valve repair'' OR ''TAVR'') AND (''male'' OR ''female'' OR "Gender disparities'' OR '' sex related outcomes") AND (''Trans catheter aortic valve replacement'' OR ''TAVR'' OR ''TAVI") AND (''Aortic valve dysfunction'' OR "aortic stenosis")	2300
Cochrane	Aortic Stenosis in All Text AND (Trans Catheter Aortic Valve Repair OR Trans Catheter Aortic Valve Replacement prosthetic valve replacement OR BPVR) in All Text AND (Gender Disparities OR Sex Based Differences OR Male OR Female) in All Text AND (TAVR OR TAVI)	120
